# Effects of Qilin pills on spermatogenesis, reproductive hormones, oxidative stress, and the TSSK2 gene in a rat model of oligoasthenospermia

**DOI:** 10.1186/s12906-019-2799-7

**Published:** 2020-02-11

**Authors:** Kaishu Zhang, Longlong Fu, Qi An, Weihong Hu, Jianxin Liu, Xiuming Tang, Yu Ding, Wenhong Lu, Xiaowei Liang, Xuejun Shang, Yiqun Gu

**Affiliations:** 1 Department of Reproductive Medicine, the Affiliated Hospital of Qingdao University, Qingdao, 266000 China; 2 National Health and Family Planning Key Laboratory of Male Reproductive Health, Department of Male Clinical Research, National Research Institute for Family Planning & WHO Collaborating Center for Research in Human Reproduction, Beijing, 100081 China; 3 Chinese Academy of Medical Sciences, Graduate School of Peking Union Medical College, Beijing, 100730 China; 4 Department of Andrology, Jinling Hospital Affiliated to Southern Medical University, Nanjing, 210002 China

**Keywords:** Traditional Chinese medicine, Qilin pills, Male infertility, Tripterygium glycosides, TSSK2

## Abstract

**Background:**

Qilin pills (QLPs), a classic Traditional Chinese Medicine (TCM) formula for treating male infertility, effectively improve semen quality in clinical trials. This study was designed to evaluate the effects of QLPs on spermatogenesis, reproductive hormones, oxidative stress, and the testis-specific serinekinase-2 (TSSK2) gene in a rat model of oligoasthenospermia.

**Methods:**

Forty adult male Sprague-Dawley (SD) rats were randomly divided into four groups. The rat model with oligoasthenospermia was generated by intragastric administration of tripterygium glycosides (TGs) once daily for 4 weeks. Then, two treatment groups were given different doses (1.62 g/kg and 3.24 g/kg) of QLPs once daily for 60 days. Sperm parameters, testicular histology and reproductive hormone measurements, oxidative stress tests, and TSSK2 expression tests were carried out.

**Results:**

QLPs effectively improved semen parameters and testicular histology; restored the levels of FSH, LH, PRL, fT, and SHBG; reduced the levels of oxidative stress products (ROS and MDA); increased testicular SOD activity; and restored the expression of spermatogenesis-related gene TSSK2.

**Conclusion:**

QLPs have a therapeutic effect on a rat model of oligoasthenospermia, and this effect is manifested as improvement of semen quality and testis histology, gonadal axis stability, decreased oxidative stress, and the regulation of testis-specific spermatogenesis-related gene TSSK2.

## Background

Human fertility has declined markedly due to various factors, such as ecological environmental pollution, an increase in work-related stress, unhealthy living habits, and sexually transmitted diseases [1–4]. The prevalence of infertility ranges from 10 to 15% among couples. Male factors are either directly or indirectly involved in approximately 50% of infertility cases [5, 6]. Oligoasthenospermia is the most common phenotype of male infertility in the clinic. The treatment of idiopathic oligoasthenospermia is mainly centered on experience-based therapeutic approaches, such as antioxidant and energy supplementation, which have limitations. Traditional Chinese Medicine (TCM) has certain advantages and characteristics in the treatment of idiopathic oligoasthenospermia [7, 8].

Qilin pills (QLPs), which are extensively used in China to treat men with oligoasthenospermia, especially idiopathic oligoasthenospermia, are a classic formula in TCM and contain 15 types of Chinese herbal medicines [9, 10]. The clinical efficacy of QLPs in the treatment of idiopathic oligoasthenospermia has recently been confirmed by two multicenter randomized controlled clinical trials (RCTs) [11, 12] and a meta-analysis [13]. Clinical observation has shown that QLPs can effectively improve semen quality and increase the pregnancy rate. Our previous animal-based study on protective effect of QLPs has also revealed that QLPs can significantly improve sperm concentration and motility and restore the testicular histology of rats with oligoasthenospermia [14, 15].

Many factors, such as urogenital infections, endocrine disorders, immunological factors and drug-related damage, affect the male reproductive system and lead to infertility [1, 2]. The specific mechanisms underlying the effects of these factors on male infertility may include spermatogenesis, hormone regulation, oxidative stress, and the regulation of spermatogenesis-related genes [16, 17].

Tripterygium glycosides (TGs) have been used to model spermatogenesis disorders in animals since 1980s in China [18]. Studies have shown that the antifertility effects of TGs are related to dysfunction of sperm cells, Sertoli cells, Leydig cells and spermatogenesis related genes [19, 20]. Ma et al. have explored the optimum dosage and time for establishing spermatogenic dysfunction rat model, with sperm concentration and motility, and pathological changes of testicular tissue used as evaluation criteria [21].

In this study, we designed an animal-based analysis to evaluate the therapeutic effects of QLPs on spermatogenesis, reproductive hormones, oxidative stress, and testis-specific serine kinase-2 (TSSK2) in a rat model of oligoasthenospermia.

## Methods

### Materials

QLPs were purchased from Guangdong Tai’antang Pharmaceutical Co., Ltd. (Guangdong, China). Chinese and Latin names of all herbal ingredients of QLPs were listed in Table 1. TGs tablets were purchased from Shanghai Fudan Fuhua Pharmacy Co., Ltd. (Shanghai, China). Testosterone (T), estradiol (E_2_), luteinizing hormone (LH), follicle stimulating hormone (FSH), free testosterone (fT), sex hormone binding globulin (SHBG), and prolactin (PRL) radioimmunoassay kits were purchased from Beijing Biosino Biotechnology and Science Incorporate (Beijing, China). Malondialdehyde (MDA), reactive oxygen species (ROS) and superoxide dismutase (SOD) assay kits were purchased from Nanjing Jiancheng Bioengineering Institute (Nanjing, China).

**Table 1:** All herbal ingredients of QLPs

Chinese name	Latin name
Zhi-He-Shou-Wu	*Polygonum multijiorum* Thunb.
Mo-Han-Lian	*Herba Ecliptae Eclipta prostrala* L.
Yin-Yang-Huo	*Epimedium brevicornu* Maxim.
Tu-Si-Zi	*Cuscuta chinensis* Lam.
Suo-Yang	*Cynomorium songaricum* Rupr.
Dang-Shen	*Codonopsis pilosula* (Franch.) Nannf.
Yu-Jin	*Curcuma aromatica* Salisb.
Gou-Qi-Zi	*Lycium chinense* Mill.
Fu-Pen-Zi	*Rubus idaeus* Linn.
Shan-Yao	*Dioscorea oppositifolia* L.
Dan-Shen	*Salvia miltiorrhiza* Bunge.
Huang-Qi	*Astragalus membranaceus* (Fisch.) Bge.
Sao-Yao	*Paeonia lactiflora* Pall.
Qing-Pi	*Citrus reticulata* Blanco.
Sang-Shen	*Morus alba* L.

### Animals

A total of 40, 8-week-old, Sprague-Dawley (SD) male rats weighing 270 ± 10 g were obtained from Vital River Laboratory Animal Technology Co., Ltd. (Beijing, China). The rats were acclimatized to standard housing conditions, including ambient temperature of 23 ± 2 °C, relative humidity at 60% ± 5%, and a 12-h light-dark cycle, in plastic cages (50 cm*35 cm*20 cm) for 1 week before initiation of the experiment. The rats were housed five per cage. The animals had free access to standard rodent chow and filtered water. The experimental protocols and ethics were approved by the Institutional Animal Care and Use Committee of the National Research Institute for Family Planning. All experiments were conducted with an effort to minimize the number of animals used and the physiological stress caused by the procedures employed.

### Oligoasthenospermia model

The rat model of oligoasthenospermia was established according to previous experiments [21] by intragastrical administration of TGs once daily for 4 weeks at a dose of 40 mg/kg/d. The rats exhibited the characteristics of oligoasthenospermia in terms of testicular pathology and sperm concentration and motility [18, 21].

### Experimental groups, treatment, and sample preparation

After the adaptation period, the animals were randomly divided into four groups containing 10 rats each. Physiological saline was continuously administered in the control group. The other 3 groups comprised the model control, low-dose QLPs, and high-dose QLPs groups. These groups were first treated with TGs to induce oligoasthenospermia, followed by physiological saline in the model control group, 1.62 g/kg QLPs (equivalent to the daily oral dose for patients based on body surface area) in the low-dose QLPs group, and 3.24 g/kg QLPs (double the low-dose QLPs treatment) in the high-dose QLPs group once daily for 60 days (equivalent to a cycle of spermatogenesis and maturation of rats). After the final treatment, the rats were weighed and anesthetized with CO_2_, and their testes and epididymides were removed by laparotomy and weighed. All efforts were made to minimize animals suffering, and euthanasia was performed by CO_2_ inhalation. The left testis of each animal was fixed in Bouin’s solution for immunohistochemical examination and the right testis was snap frozen in liquid nitrogen and stored at − 80 °C until the oxidative stress parameters, namely, the levels of MDA, ROS, and SOD, were measured. The blood serum was obtained by centrifugation (1500 rpm, 15 min, 4 °C) and stored at − 80 °C until use for biochemical determinations.

### Analysis of sperm concentration and motility

The whole left epididymis of each rat was harvested immediately after sacrifice and cut into small pieces that were transferred to a tube containing 2 mL of warm (37 °C) phosphate-buffered saline (PBS) and 1 mL Medium 199 (Sigma, USA), which was shaken at 37 °C for 15 min to allow induce the sperm to swim. Then, 10 μL of diluted sperm suspension were transferred to each counting chamber of the hemocytometer to determine sperm concentration and motility which was measured as the percentage of motile sperm (a + b grade) in total spermatozoa [22].

### Histopathological analysis

Testis tissue was fixed in Bouin’s solution for 48 h, routinely processed with an automatic tissue processor, dehydrated, embedded in paraffin, sectioned at 5-μm, and stained with hematoxylin and eosin (H&E). Cell morphology was observed under a light microscope (Nikon Eclipse TS100, Japan) and evaluated with Johnsen scoring [23]. The histological criteria for modified Johnsen scoring are as follows: full spermatogenesis (score 10), slightly impaired spermatogenesis, many late spermatids, disorganized epithelium (score 9), less than five spermatozoa per tubule, few late spermatids (score 8), no spermatozoa, no late spermatids, many early spermatids (score 7), no spermatozoa, no late spermatids, few early spermatids (score 6), no spermatozoa or spermatids, many spermatocytes (score 5), no spermatozoa or spermatids, few spermatocytes (score 4), spermatogonia only (score 3), no germinal cells, Sertoli cells only (score 2), and no seminiferous epithelium (score 1).

### Detection of reproductive hormones

Serum stored at − 80 °C was thawed at room temperature. The hormonal analyses were performed using commercially available kits and in accordance with the manufacturer’s instructions.

### Oxidative stress in testes

In view of the role of oxidative stress in male infertility, ROS, SOD, and MDA levels in the testes were tested [24, 25]. The testicular tissues were homogenized in 10× ice-cold PBS and centrifuged at 4000 rpm for 15 min. The supernatant were used to determine the ROS, SOD, and MDA levels in the testes, and these levels were determined using commercially available kits, in accordance with the manufacturer’s instructions.

### qRT-PCR

The mRNA levels of spermatogenesis-related gene TSSK2 was determined by qRT-PCR. Total RNA was isolated using the TRIzol reagent (Invitrogen) according to the manufacturer’s instructions. The quality of extracted RNA was verified by agarose gel electrophoresis and used to synthesize cDNA using the PrimeScript RT reagent kit with gDNA Eraser (Takara Bio, Japan). The sequences of primers are the following: TSSK2: forward primer 5′-CCGCAAGAAAACACCCACT-3′, reverse primer 5′-CTCGGCACTTGATGAACTCG-3′; GAPDH: forward primer 5′-TTCCTACC CCCAATGTATCCG-3′; reverse primer 5′-CCACCCTGTTGCTGTAGCCATA-3′. The thermal cycling conditions were 6 min at 95 °C, followed by 40 cycles of denaturation at 95 °C for 10 s, annealing at 58 °C for 10 s, and extension at 72 °C for 30 s. The expression levels of the mRNAs of each sample were normalized, with GAPDH serving as an internal control. The results were expressed as relative expression ratios with respect to the control group. The specificity of each primer was assessed by melting curve analysis. Data were analyzed with the 2^−ΔΔCt^ method. All RT-PCRs were performed in triplicate and the data were presented as mean ± SD.

### Western blot

Equal quantities of protein from the testis tissue lysate were processed for Western blotting (Roche, USA). Each sample was denatured, electrophoresed, and transferred onto a polyvinylidenedifluoride membrane. Specific steps were as follows: proteins were resolved by 10% sodium dodecyl sulfate–polyacrylamide gel electrophoresis after denaturation at 95 °C for 5 min, and transferred to a polyvinylidenedifluoride membrane that was blocked overnight at 4 °C in PBS containing 0.1% Tween 20 (PBS-T) and 5% skim milk. The primary antibody used was rabbit anti-rat TSSK2 (1:500, Abcam, USA). After three washes with TBS-T, the membrane was incubated at room temperature for 1 h with horseradish peroxidase (HRP)-conjugated goat anti-rabbit secondary antibody (1:1000; Cell Signaling Technology) in PBS-T with 2% skim milk and washed three times with TBS-T. The blots were visualized with LumiGLO reagent and peroxide, followed by exposure to X-ray film. Western blot analyses were performed at least in triplicate.

### Immunohistochemistry

Rat testis tissue was fixed by soaking in Bouin fixing fluid at room temperature for 48 h, and they then were dehydrated, embedded in paraffin, and cut into 5-μm-thick sections that were collected on glass slides. The sections were deparaffinized in xylene, rehydrated through a graded series of ethanol, and rinsed with water. Endogenous peroxidase activity was blocked by incubation in 0.3% hydrogen peroxide in PBS for 30 min at room temperature. The slides were blocked for 1 h in PBS supplemented with 10% normal goat serum. TSSK2 expression was detected after overnight incubation at 4 °C with antibodies against TSSK2 (1:200, Abcam, USA). After washing, the sections were incubated for 1 h with HRP-conjugated goat anti-rabbit IgG (1:2000; Santa Cruz Biotechnology, USA) in 10% goat serum, counterstained for 10s with hematoxylin (Gill no. 3; Sigma, USA). Imaging analyses was conducted using a confocal microscope (Nikon Eclipse TS100, Japan).

### Statistical analysis

Data in accordance with the normal distribution are expressed as the mean ± standard deviation and were analyzed with SPSS software (version 22.0; Chicago, USA). Differences between group means were assessed by one-way analysis of variance (ANOVA) followed by the Student-Newman-Keuls test. *P* < 0.05 was considered statistically significant.

## Results

### General situation of experimental rats

In general, the rats in each group took normal food and water during the modeling and administration period. The consumption of food and water in rats increased gradually with body weight. There were no toxic symptoms such as nausea, sleepiness, sluggish reaction, hypokinesia, shedding of body hair, or obvious wasting. No rat died during modeling and administration.

### QLPs streatment improves sperm quality

Results of semen parameters were shown in our previous paper [14]. Sperm concentration and motility were lower in model group than in control group (*P* < 0.05), indicating the successful establishment of the oligoasthenospermia rat model. Compared with the model group, sperm concentration and motility were increased by QLPs administration (*P* < 0.05). Sperm concentration but not sperm motility was higher in high-dose QLPs group than in low-dose QLPs group (*P* < 0.05).

### QLPs treatment reverses histopathological damage

**Fig. 1:**
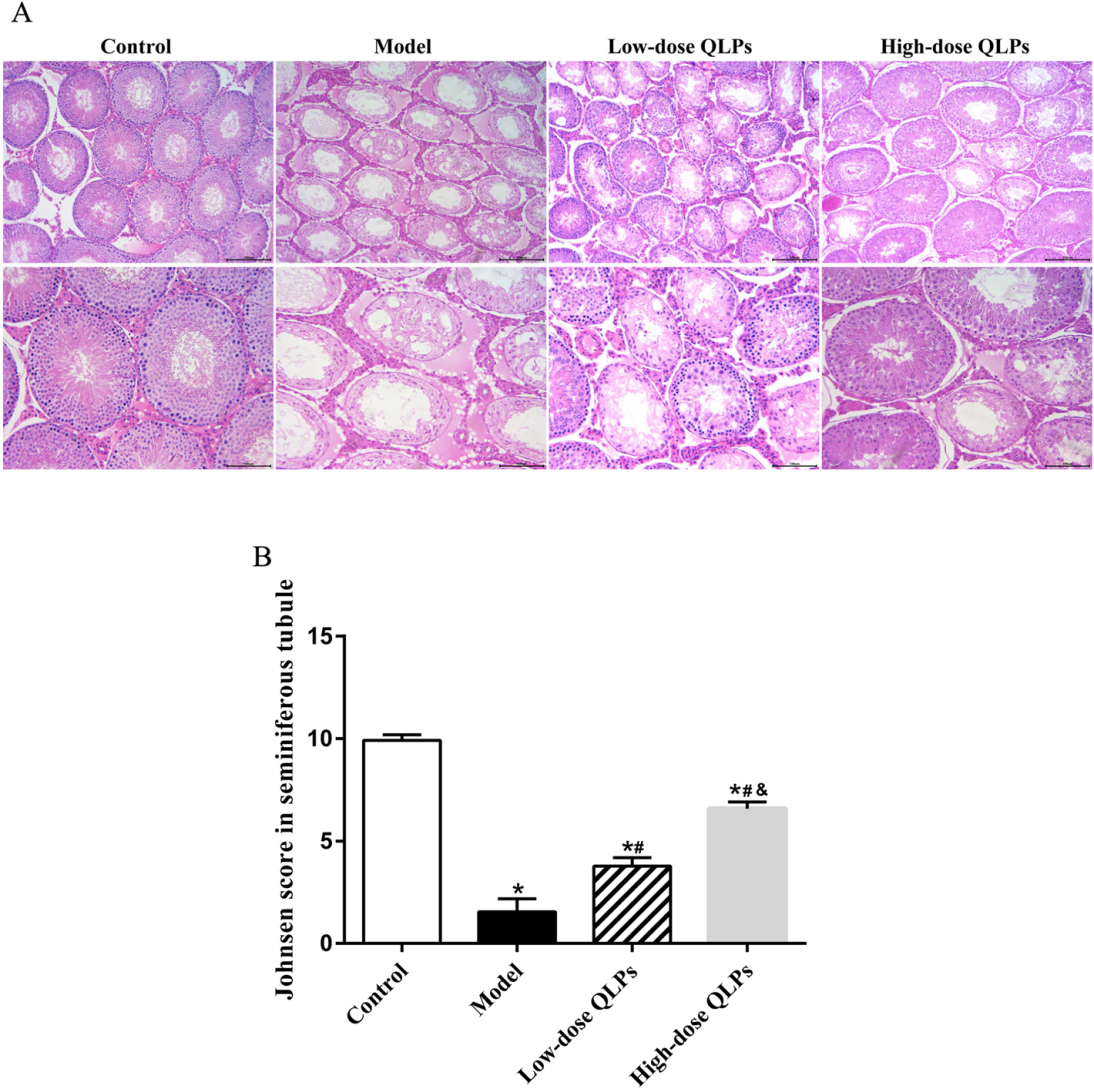
HE staining of testicular tissues and Johnsen scoring among groups. **a** The top line: magnification× 100; the bottom line: magnification× 200. Control group with normal histology of seminiferous tubules and interstitium. Oligoasthenospermia model group with loss of spermatogenic cells, degeneration of interstitial cells, and vacuolization in the cytoplasm of Sertoli cells. Low-dose QLPs group with increased numbers of spermatogenic cells, hyperplasia of interstitial cells, and decreased number of vacuoles in Sertoli cells. High-dose QLPs group with further enhancement of tissue recovery as compared to the low QLPs dose group. **b** Significant difference was found in Johnsen scoring among groups. (^*^P < 0.05 versus the control group; ^#^*P* < 0.05 versus the model group; ^&^*P* < 0.05 versus the low-dose QLPs group)

The cross section of testicular tissue is shown in Fig. 1a. It mainly includes the seminiferous tubules and the interstitium between tubules. Seminiferous tubules are the place where spermatozoa are produced, which are composed of Sertoli cells, spermatogonia, spermatocytes and spermatids. And interstitial tissue mainly includes blood vessels, lymphatic vessels, fibroblasts, macrophages, columnar cells and Leydig cells [26]. A histological analysis of testicular tissue from control group revealed a normal process of spermatogenesis, with a regular arrangement of spermatogenic epithelial cells in the seminiferous tubules. In contrast, the model group exhibited testicular damage including loss, disorganization, and sloughing of spermatogenic cells, degeneration of interstitial cells, and vacuolization in the cytoplasm of Sertoli cells, which were consistent with oligospermia. QLPs administration partly restored the morphology of Leydig, Sertoli, and spermatogenic cells, with the most dramatic improvement observed in high-dose QLPs group (Fig. 1a). There were significant differences in Johnsen scoring among groups (Fig. 1b).

### QLPs treatment regulates reproductive hormones

The concentrations of serum FSH, LH, PRL, fT, and SHBG were significantly higher in the model control group than in the other groups, while the values of the low-dose QLPs group were higher than those of the model group (Table. 2). However, no significant differences were evident between the concentrations of serum T in the groups, though the concentration of serum T was lower in the model group than in the other groups. Similarly, the E_2_ concentration was lower in the control groups than in the model and QLPs administration groups, and higher in the low-dose and high-dose QLPs groups than in the model group (Table. 2).

**Table 2:** Effects of QLPs on sex hormone level of the different groups of rats

Group	n	FSH (mIU/ml)	LH (mIU/ml)	PRL (mIU/ml)	E_2_ (pg/ml)	T (ng/ml)	fT (nmol/l)	SHBG (nmol/l)
Control	10	1.35 ± 0.58	2.87 ± 0.63	56.84 ± 24.86	4.28 ± 1.52	0.66 ± 0.35	23.88 ± 2.48	74.10 ± 9.67
Model	10	1.93 ± 0.62^*^	3.89 ± 0.93^*^	128.28 ± 38.20^*^	7.06 ± 2.93^*^	0.48 ± 0.24	27.42 ± 2.17^*^	82.36 ± 10.19^*^
Low-dose QLPs	10	1.28 ± 0.52^#^	2.97 ± 0.69^#^	44.82 ± 17.10^#^	11.97 ± 3.48^*#^	0.64 ± 0.28	18.92 ± 2.04^*#^	65.87 ± 6.83^*#^
High-dose QLPs	10	1.62 ± 0.61	3.49 ± 0.68	46.91 ± 30.95^#^	12.26 ± 1.77^*#^	0.73 ± 0.27	19.76 ± 2.83^*#^	70.35 ± 6.09^*#^

^*^: *P* < 0.05 versus the control group; ^#^: *P* < 0.05 versus the model group; ^&^: *P* < 0.05 versus the low-dose QLPs group

### QLPs treatment decreases oxidative stress

As shown in Table 3, the model rats pretreated with TGs alone showed high levels of ROS and MDA and a low level of SOD compared to the levels in the control group. The levels of SOD in the testes were significantly increased in the QLPs-treated group compared to the control group (*P* < 0.05; Table. 3). The activities of ROS and MDA increased in the TGs-treated group but decreased in the QLPs-treated group compared with the activities in the control group. However, compared to the model group, the ROS levels in the QLPs-treated groups were significantly decreased, and the SOD activities were increased.

**Table 3:** Comparison of oxidative stress indexes in rat testes

Group	n	SOD (U/ml)	ROS (IU/ml)	MDA (nmol/ml)
Control	10	538.07 ± 84.18	195.58 ± 46.08	1.46 ± 0.37
Model	10	412.75 ± 68.77^*^	432.02 ± 31.48^*^	1.93 ± 0.27^*^
Low-dose QLPs	10	570.90 ± 102.84^#^	386.31 ± 22.75^*#^	1.86 ± 0.31
High-dose QLPs	10	560.38 ± 81.29^#^	255.01 ± 37.60^*#&^	1.52 ± 0.38^#^

^*^: *P* < 0.05 versus the control group; ^#^: *P* < 0.05 versus the model group

^&^: *P* < 0.05 versus the low-dose QLPs group

### QLPs treatment recovers the mRNA level of TSSK2

**Fig. 2:**
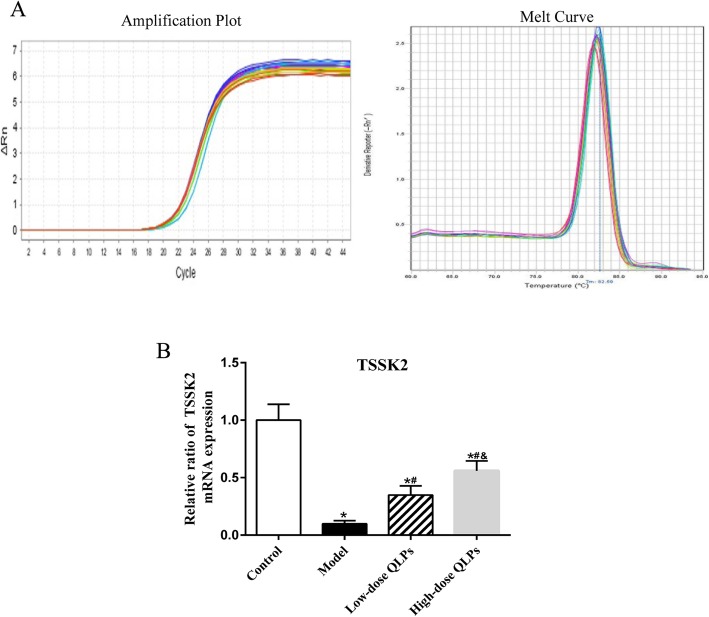
Relative mRNA levels of spermatogenesis-related genes TSSK2. **a** Amplification plot and melting curve. **b** TSSK2 mRNA expression was sharply down-regulated in the oligoasthenospermia model group, while QLPs treatment reversed it. And the increase of TSSK2mRNA expression was more obvious in high-dose group. (^*^P < 0.05 versus the control group; ^#^*P* < 0.05 versus the model group; ^&^*P* < 0.05 versus the low-dose QLPs group)

Given the regulation effect of TSSK2 gene on spermatogenesis, qRT-PCR analysis was carried out to assess whether QLPs can modulate TSSK2 gene expression (Fig. 2a). The results show that the mRNA level of TSSK2 decreased in the model control group with oligoasthenospermia. Thus QLPs reversed the mRNA level of TSSK2 in treatment groups. Meantime, expression of TSSK2 at the mRNA level was significantly changed in high-dose QLPs group compared to low-dose QLPs group (Fig. 2b).

### QLPs treatment restores protein expression of TSSK2

**Fig. 3:**
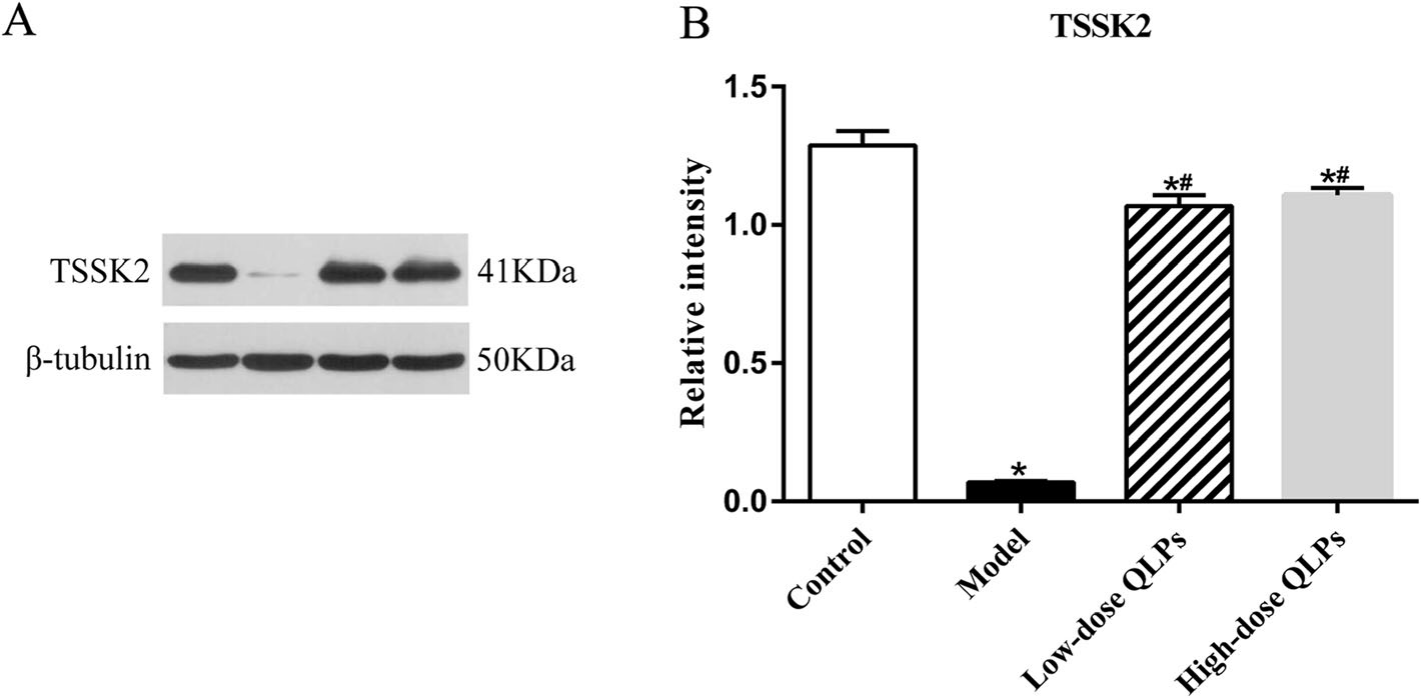
Western blot analysis of TSSK2 protein expression. **a** Equal amounts of protein from the testis tissue lysates were analyzed for the expression of indicated proteins. **b** TSSK2 was sharply down-regulated in the oligoasthenospermia model control group, and this effect was reversed by the QLPs treatments. (^*^P < 0.05 versus the control group; ^#^*P* < 0.05 versus the model group; ^&^*P* < 0.05 versus the low-dose QLPs group)

Western blot analysis of protein expression showed that the TSSK2 levels were decreased in the model control group with oligoasthenospermia. However, this effect was reversed by the QLP treatments, suggesting QLP-mediated TSSK2 gene activation in the rat testes (Fig. 3a, b).

### Immunohistochemistry for the localization of TSSK2

**Fig. 4:**
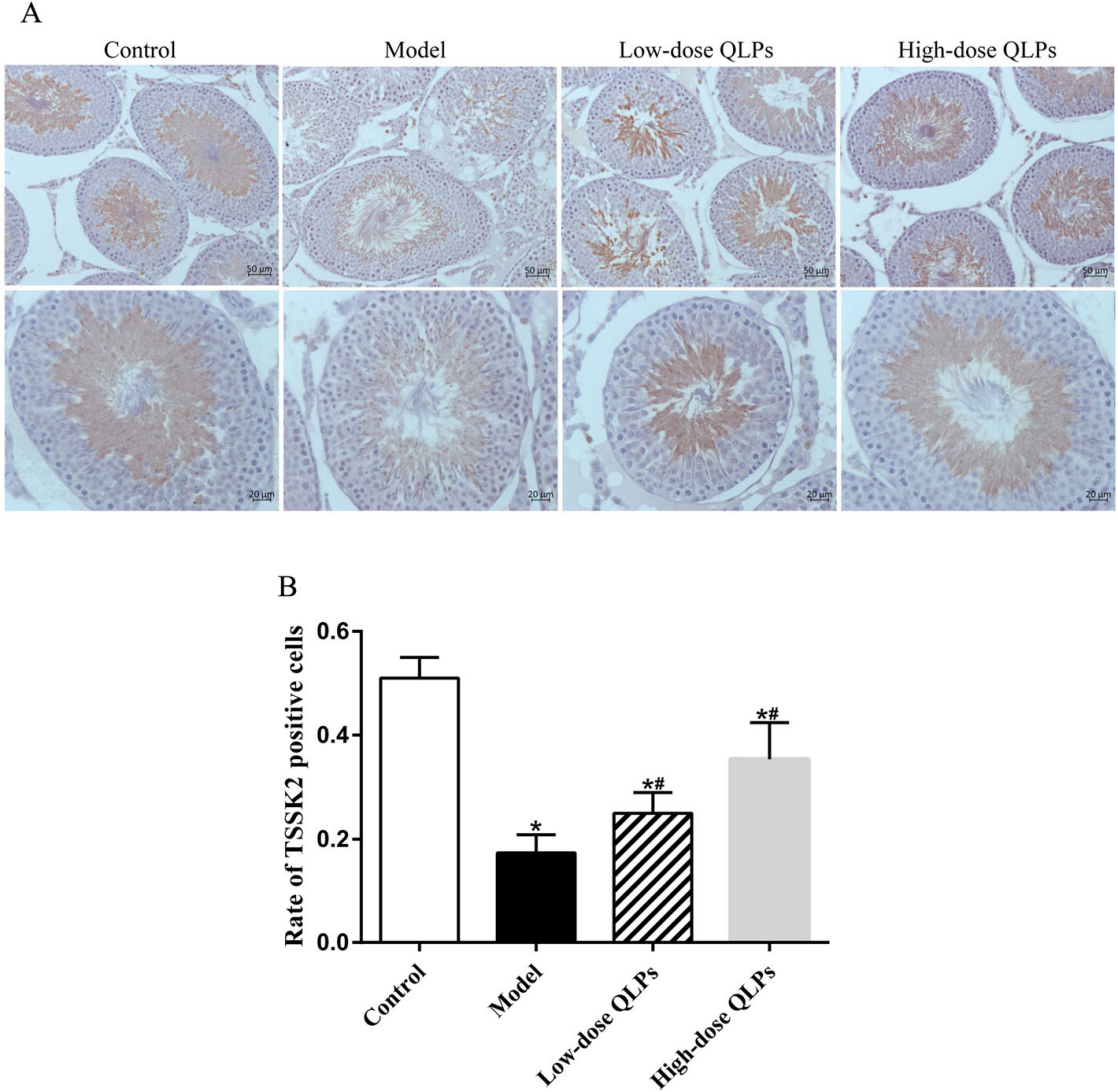
Expression and location of TSSK2 proteins in testis tissue. **a** The top line: magnification× 200; the bottom line: magnification× 400. TSSK2 expression was detected by immunohistochemistry. TSSK2 was expressed in spermatids and spermatogonia. **b** TSSK2 expression was lower in the model control group with oligoasthenospermia than in the control and QLPs-treated groups. (^*^P < 0.05 versus the control group; ^#^*P* < 0.05 versus the model group; ^&^*P* < 0.05 versus the low-dose QLPs group)

The TSSK2 protein was expressed in spermatids and spermatogonia (Fig. 4a). TSSK2 immunoreactivity was lowest in the model control group and increased in a dose-dependent manner after QLP administration (Fig. 4b).

## Discussion

In previous study, we confirmed that QLPs could clearly alleviate TGs-induced reproductive system damage by improving sperm quality and the histology of rat testis, effectively protecting reproductive function [14]. The therapeutic effect of QLPs on spermatogenesis function was confirmed in the present experiment also by improving sperm quality and testis histology. Sperm concentration and motility were increased by low- and high-dose QLP administration relative to oligoasthenospermia model group. QLP administration partly restored the morphology of Leydig, Sertoli, and spermatogenic cells damaged by TGs. Since studies have shown that spermatogenesis and maturation are regulated by the gonadal axis, affected by oxidative stress and spermatogenesis-related genes [27–30], the effects of QLPs on reproductive hormones, oxidative stress and spermatogenesis-related gene TSSK2 were evaluated in the present study.

In the present study, we used TGs to establish a rat model of oligoasthenospermia, which reflected the histopathological changes in the testis, abnormal sperm morphology, and reduced sperm motility. TGs induced atrophy of contorted seminiferous tubules, thinning of the seminiferous epithelium, and reduced the number of spermatogenic cells, resulting in decreased sperm concentration and motility sperm in the epididymis. These changes related to necrosis and aseptic inflammation was consistent with the study carried out by Ma HF [21]. QLPs administration partly restored the morphology of Leydig, Sertoli, and spermatogenic cells, which was the base of spermatogenesis. It was also verified by data gained from our previous study about the protective effect of QLPs on the reproductive function [14]. Johnsen scoring was used to assess the change of testicular histology in the present study. Significant difference was found among groups, which revealed the success of tablishing spermatogenic dysfunction rat model by TGs and the therapeutical effect of QLPs on pathologic testis.

Reproductive hormone, especially serum FSH, LH, and T, plays roles in the maintenance of male reproductive function. Testicular activity is governed by testicular T and gonadotrophic hormones (FSH and LH). LH stimulates the release of T from Leydig cells, and FSH regulates the production of spermatozoa by acting on Sertoli cells [31]. In this study, increased serum FSH and LH levels were observed in the TGs-induced model control group. However, there was no significant difference in FSH and LH levels between the high dose QLP group and the model group. Possible reasons for this were as follows. Due to the damage effect of TGs, spermatogenic function was impaired, which caused the regulation of gonadal axis to make FSH and LH elevated in model group. In the high-dose QLPs group, QLPs itself positively regulated hormones and increased the level of FSH and LH.

Although the serum T levels exhibited a downward trend without statistical significance, the results revealed the effect of TGs on the gonadal axis. The analysis showed that the serum LH, FSH and T levels remained normal after the administration of QLPs, indicating that QLPs could restore the balance of the gonadal axis. A normal gonadal axis is important for the recovery and maintenance of spermatogenesis. A compensatory increase in serum LH levels directly stimulated T production (manifested as the increased level of fT) from Leydig cells in the model control group of our studies, which was consistent with the study by Wisniewski P [32]. As shown in previously published data, a decrease in serum T levels is related to the severity of spermatogenic cell damage, disordered spermatogenesis and a decline in sperm quality and can therefore cause oligoasthenospermia [33].

Under physiological conditions, PRL and LH stimulate the production of steroids. Elevated prolactin in a short term may promote the secretion of ketones, but long-term hyperprolactinemia can reduce the production of ketones and destroy sperm production [34]. In animal models, PRL also regulates spermatogenesis. PRL induces the expression of FSH in Sertoli cells and stimulates the progression of germ cells from spermatocyte to spermatid [35]. In the model control group of this study, PRL increased significantly and positively regulated FSH to a higher level, resulting in disordered spermatogenesis. After treatment with QLPs, the levels of serum PRL and FSH in the damaged rats gradually recovered.

Testicular T concentrations play a central role in maintaining normal spermatogenesis. Low testicular T levels can impair spermatogenesis. However, elevated E_2_ levels inhibit pituitary gonadotropin secretion, resulting in down-regulation of Leydig cell function, decreased T production and decreased T levels in both the testis and serum [36]. The balance between serum androgen and estrogen levels is essential for maintaining normal spermatogenesis by means of the serum levels of SHBG, which transports androgens and estrogens in blood and regulates steroid access to target tissues. In this study, the levels of serum SHBG in the QLP treatment groups were decreased, which together with higher androgen levels, played the role of T pool, thereby maintaining the stability of biologically active serum T levels and ensuring the spermatogenesis process.

Some male infertility patients with severe spermatogenesis impairments present with strong aromatase activity, which is characterized by relatively low serum T levels and elevated E_2_ levels [37, 38]. In the model control group of the present study, relatively decreased T levels were due to the decreased synthesis of testicular T in the testis or to increased metabolic clearance of serum T. Increased metabolic clearance of serum T by the stimulation of aromatase, a key enzyme in the conversion of T to E_2_, leads to an increase in E_2_ levels. The statistically significant increase in the E_2_ levels of the QLP treatment groups in our study are not consistent with the observations of previous studies [14], and this discrepancy may be related to excessive aromatase enzyme activity during spermatogenesis.

By examining the influence of the gonadal axis, we found that QLPs could significantly restore the levels of sex hormone, except for E_2_ and fT. The balancing effects of QLPs on reproductive hormones were helpful toward maintaining normal spermatogenesis, sperm concentrations and sperm vitality. Based on the observation of a spermatogenic cycle in the rats after QLP treatment, during which the recovery of pituitary hormones was faster than that of testicular hormones, the results failed to fully reflect the treatment effects of QLPs.

Oxidative stress, which disrupts the steady state relationship between the production of ROS and the antioxidant defensive capacity of the body, is an important factor that contributes to the loss of sperm motility and to male infertility [39]. Under physiological conditions, ROS are formed during oxygen metabolism, and ROS concentrations are controlled by antioxidant defense mechanisms, such as SOD. However, the overproduction of ROS may result in oxidative stress that has a significant adverse impact on semen quality and male fertility [40]. Increased oxidative stress causes a decrease in intracellular ATP levels and the release of apoptogenic factors (pro-caspase cytochrome C, apoptosis-inducing factors) into the cytosol as a result of mitochondrial membrane disruption, enzyme dysfunction, protein phosphorylation disruption, increased membrane permeability, and spermicidal products formation, thereby decreasing semen quality [39]. The spermatogenic cell membrane and sperm cells are very susceptible to attack by ROS-mediated oxidative damage since these components are rich in polyunsaturated fatty acids, and this damage may result in decreased sperm motility [40].

Since the generation of low levels of ROS is an important component of the signal-transduction-stimulating capacity of spermatozoa [41], excessive ROS levels induce lipid peroxidation of the sperm cell membrane, the malfunction of capacitation, impaired acrosome reactions, and a loss of motility [42]. Lipid oxidation products, including MDA, are reliable biomarkers of oxidative stress [43]. In the current study, TG-induced reproductive toxicity was associated with elevated oxidative stress in testes, as evidenced by the increased levels of testicular ROS and MDA.

Increased lipid peroxidation and altered membrane function can affect sperm motility and cause sperm dysfunction, which may be a consequence of a rapid loss of intracellular ATP leading to decreased sperm viability [44]. ROS is capable of disrupting the androgen-producing Leydig cells and may cause increased lipid peroxidation and DNA fragmentation in germ cells. Antioxidant enzymes provide the first line of defense against the deleterious effects of ROS [45]. SOD catalyzes the dismutation of superoxide radicals to hydrogen peroxide (H_2_O_2_) and molecular oxygen, whereas catalase (CAT) and glutathioneperoxidase (GSH-PX) are responsible for H_2_O_2_ detoxification [46]. The decreased activity of SOD leads to the increased production of MDA via the catalytic cracking of lipid peroxides in the presence of metal ions. MDA is toxic to cells and can form intramolecular and intermolecular cross-linkages with proteins to induce apoptosis [47].

In the present investigation, the testes of the TGs-treated rats showed significantly decreased testicular SOD activity, which further led to significantly increased lipidperoxidation in the testes. Lipid peroxidation is one of the primary processes that result from oxidative stress. The QLP treatments resulted in enhanced antioxidative enzyme activities, thereby suppressing lipid peroxidation and thus rescuing the testes from the TGs-induced oxidative load. In summary, compared to the model group, the ROS levels in the QLPs-treated groups were significantly decreased, and the SOD activities were increased. These results suggested that the oxidative stress products were effectively scavenged after QLP treatments and that the treatment improved the testes antioxidant ability.

Spermatogenesis is a complex process involving specific interactions between the developing germ cells and their support cells, namely, Sertoli cells, within the seminiferous tubules. This process is regulated by androgen-producing Leydig cells, which are located in the interstitial tissue surrounding the seminiferous tubules. The molecular mechanisms regulating spermatogenesis are largely unknown; however, several kinases have been implicated in various stages of spermatogenesis, primarily in the control of meiosis [48]. TSSK2, a member of the TSSK family, is expressed exclusively during the cytodifferentiation of late spermatids tosperms [49, 50]. Therefore, TSSK2, a specific phosphorylated protein of testicular tissue, can be used to detect spermatogenesis in testicular tissue. In our study, the expression of TSSK2 was examined in each group by qRT-PCR, western blot and immunohistochemistry. TSSK2 expression in the model control group was significantly weakened. However, TSSK2 expression gradually recovered after the treatments with QLPs, indirectly demonstrating the role of QLPs in regulating the expression of spermatogenic genes.

The specific mechanism by which QLPs regulate the reproductive system is still unclear. The effects of QLPs on semen quality, testicular pathology, reproductive hormones, oxidative stress and spermatogenesis-related gene TSSK2 were associated with the ingredients of QLPs. *Lycium chinense* Mill. plays a significant role in the recovery of serum testosterone levels, increased superoxide dismutase activity, decreased malondialdehyde levels, promoted oxidative balance and rescued testicular DNA damage [51]. *Cuscuta chinensis* Lam. increased the weights of testis, epididymis and pituitary gland, and stimulated T and LH secretion both in vitro and in immature rats [52]. And *Epimedium brevicornu* Maxim. exerted beneficially protective effects on the structural and functional damage of male mice reproductive system and reduced apoptosis in spermatogenic cells by inhibiting oxidative stress [53]. The protection and regulatory role of QLPs on reproductive function is the result of synergistic effect of various components of QLPs. We initially explored the possible molecular mechanism of QLPs on alleviating oligoasthenospermia [15]. Our previous studies revealed that the improvement function of QLPs on sperm and testis works mainly by suppressing mitochondrial apoptosis in the testis via modulation of B cell lymphoma (Bcl)-2, Bcl-2-associated X protein (Bax), cytochrome C, caspase-9 and caspase-3 expression. But beyond that, other specific mechanism of the QLPs on the reproductive system still needs further research.

## Conclusions

QLPs have effects on the entire spermatogenesis process, and these effects are not only manifested in maintaining the balance of reproductive hormones but also in reducing oxidative stress, which can inhibit spermatogenic cell apoptosis. In addition, QLPs also have regulatory effects on spermatogenesis-related genes, which directly affect the process of spermatogenesis.

## Data Availability

The datasets used and/or analyzed for this study are available from the corresponding author by reasonable request.

## Abbreviations

CAT: Catalase
E_2_: Estradiol
FSH: Follicle stimulating hormone
fT: Free testosterone
GAPDH: Glyceraldehyde 3-phosphate dehydrogenase
GSH-PX: Glutathioneperoxidase
H_2_O_2_: Hydrogen peroxide
LH: Luteinizing hormone
MDA: Malondialdehyde
PBS: Phosphate-buffered saline
PRL: Prolactin
QLP: Qilin pill
RCT: Randomized controlled clinical trial
ROS: Reactive oxygen species
SHBG: Sex hormone binding globulin
SOD: Superoxide dismutase
T: Testosterone
TCM: Traditional Chinese Medicine
TGs: Tripterygium glycosides
TSSK2: The testis-specific serinekinase-2
